# Antioxidative Sirt1 and the Keap1-Nrf2 Signaling Pathway Impair Inflammation and Positively Regulate Autophagy in Murine Mammary Epithelial Cells or Mammary Glands Infected with *Streptococcus uberis*

**DOI:** 10.3390/antiox13020171

**Published:** 2024-01-29

**Authors:** Sohrab Khan, Tian Wang, Eduardo R. Cobo, Bingchun Liang, Muhammad Asfandyar Khan, Maolin Xu, Weijie Qu, Jian Gao, Herman W. Barkema, John P. Kastelic, Gang Liu, Bo Han

**Affiliations:** 1 College of Veterinary Medicine, China Agricultural University, Beijing 100193, China; drsohrab@cau.edu.cn (S.K.); b20223050426@cau.edu.cn (T.W.); liangbingchun@cau.edu.cn (B.L.); asfand@cau.edu.cn (M.A.K.); xx0517@cau.edu.cn (M.X.); gaojian2016@cau.edu.cn (J.G.); 2 Faculty of Veterinary Medicine, University of Calgary, Calgary, AB T2N 4N1, Canada; ecobo@ucalgary.ca (E.R.C.); barkema@ucalgary.ca (H.W.B.); jpkastel@ucalgary.ca (J.P.K.); 3 College of Veterinary Medicine, Yunnan Agricultural University, Kunming 650201, China; wjqu@ynau.edu.cn

**Keywords:** bovine mastitis, *Streptococcus uberis*, antioxidative pathway, inflammation, autophagy, murine mammary glands, mMECs

## Abstract

*Streptococcus uberis* mastitis in cattle infects mammary epithelial cells. Although oxidative responses often remove intracellular microbes, *S. uberis* survives, but the mechanisms are not well understood. Herein, we aimed to elucidate antioxidative mechanisms during pathogenesis of *S. uberis* after isolation from clinical bovine mastitis milk samples. *S. uberis*’s in vitro pathomorphology, oxidative stress biological activities, transcription of antioxidative factors, inflammatory response cytokines, autophagosome and autophagy functions were evaluated, and in vivo *S. uberis* was injected into the fourth mammary gland nipple of each mouse to assess the infectiousness of *S. uberis* potential molecular mechanisms. The results showed that infection with *S. uberis* induced early oxidative stress and increased reactive oxygen species (ROS). However, over time, ROS concentrations decreased due to increased antioxidative activity, including total superoxide dismutase (T-SOD) and malondialdehyde (MDA) enzymes, plus transcription of antioxidative factors (Sirt1, Keap1, Nrf2, HO-1). Treatment with a ROS scavenger (N-acetyl cysteine, NAC) before infection with *S. uberis* reduced antioxidative responses and the inflammatory response, including the cytokines IL-6 and TNF-α, and the formation of the Atg5-LC3II/LC3I autophagosome. Synthesis of antioxidants determined autophagy functions, with Sirt1/Nrf2 activating autophagy in the presence of *S. uberis*. This study demonstrated the evasive mechanisms of *S. uberis* in mastitis, including suppressing inflammatory and ROS defenses by stimulating antioxidative pathways.

## 1. Introduction

Mastitis is an inflammatory syndrome that can irreversibly damage the mammary glands of dairy cattle [1], causing substantial financial losses due to reductions in milk production and quality, plus premature culling [2,3]. Bovine mastitis is often caused by various environmental and bacterial agents, including *Streptococcus uberis* [4], the most frequently isolated pathogen causing subclinical mastitis in the United Kingdom, Ireland, Australia and New Zealand [5]. The damaging consequences of *S. uberis* infection include inflammatory and autophagy responses [6] regulated by antioxidative factors [7]. Autophagy is a defensive catabolic mechanism to remove damaged organelles and control bacterial infections [8]. “Autophagy flux” refers to the entirety of autophagosome production, its maturation, fusion with lysosomes and subsequent breakdown, with release of macromolecules into the cytosol. Autolysosomes and degradation are key functions of autophagy [9].

Production of reactive oxygen species (ROS) significantly increases during infection, aids pathogen removal and contributes to inflammatory signaling cascades [10]. Production of ROS is regulated by antioxidative factors, including silent information regulator 1 (Sirt1), which reduces oxidative stress by modulating superoxide dismutase (SOD) [11] and decreases malondialdehyde (MDA), an oxidative stress marker [12]. Sirt1 also affects signaling pathways associated with inflammation and autophagy [13]. Other antioxidant effectors are Kelch-like ECH-associated protein 1 (Keap1) and Nuclear factor erythroid 2-related factor 2 (Nrf2) [14]. Under physiological conditions, Keap1 regulates Nrf2 activity in the cytoplasm and is a principal protective sensor for oxidative stress [15]. However, under oxidative stress, Nrf2 separates from Keap1 protein, moves into the nucleus and joins with antioxidant response elements to promote the production of heme oxygenase-1 (HO-1) and SOD [16,17]. Expression of inflammatory markers (namely TNF-α/IL-6) and oxidative damage are reduced by activation of the Nrf2/HO-1 signaling pathway [18,19]. Therefore, Nrf2/HO-1 is an antioxidant signal that aims to reduce inflammation provoked by infection and oxidative stress [20]. Dissociation of Nrf2 from Keap1 activates autophagy by coupling the stress-inducible p62/SQSTM1 protein complex [21]. This multifunctional protein core has multiple domains, including a Keap1-interacting region (KIR) and an LC3-interacting region (LIR) [22]. The lipidation of microtubule-associated protein 1A/1B-light chain 3 to generate LC3II is critical in promoting autophagy [23]. The protein P62 interacts with LC3 in the autophagy–lysosome pathway and targets ubiquitinated substrate cargoes for destruction [24]. Furthermore, Atg5 promotes autophagosome elongation and requires conversion of LC3I to LC3II [25].

Microbial pathogens that infect host cells can be subjected to autophagy. In this process, LC3, which is widely distributed in the cytoplasm, connects to targeted substrates [26]. Furthermore, Atg4 cuts the C-terminal portion of LC3 into LC3-I that is then activated by Atg7 (an E1-like enzyme), passed to Atg3 (an E2-like enzyme) and changed into membrane-bound LC3II. Invading pathogens with polyubiquitination are typically identified by the autophagy receptor P62 that delivers the targeted substrate to LC3II-bound membranes and facilitates autophagosome formation. Attracting autophagy-related proteins to phagocytic vesicle assembly sites, e.g., Atg5, is important in phagophore synthesis, and requires a signal for autophagy [27]. The usual outcome is containment of intracellular infections or recycling of cytosolic substances [28].

We identified autophagy as a crucial component of epithelial cell defense against mastitis pathogens, with the involvement of HIF-1α, AMPKα/ULK1 and the PTEN/PI3K-Akt-mTOR pathway [29,30,31]. Furthermore, we also reported that *S. uberis* infection induced autophagy by decreasing synthesis of pro-inflammatory cytokines [32]. However, the mechanisms that connect the antioxidative pathway and autophagy during *S. uberis* infection remain unclear. Hence, we tested the hypothesis that silencing/activation of antioxidative pathway factors will bring changes in autophagy mechanism.

Our objective was to characterize pathogenesis of *S. uberis* mastitis and the role of the antioxidative pathway in the modulation of autophagy, using murine in vitro and in vivo models of mammary epithelial cells (mMECs) and mammary glands, respectively. Specifically, we addressed antioxidative factors (Sirt1, Keap1 and Nrf2), inflammatory factors (IL-6 and TNF-α) and autophagy marker proteins (Atg5 and LC3II/LC3I). We concluded that Sirt1 and Keap1-Nrf2 were involved in the activation of autophagy by reducing T-SOD and MDA activity along with significant reductions in inflammatory responses.

## 2. Materials and Methods

### 2.1. Statement of Ethics

This study was reviewed and approved by the Ethical Committee of the College of Veterinary Medicine, China Agricultural University (CAU), Beijing, China (Protocol SYXK, 2016-0008). Furthermore, it was conducted according to standard ethical guidelines implemented at CAU.

### 2.2. S. uberis Isolation

Milk samples from dairy cows with clinical mastitis were obtained from dairy farms in northern Beijing, China, and *S. uberis* was isolated from those samples [32]. Isolates were identified on Todd–Hewitt Agar (THA), a solid medium containing 5% sheep blood, and cultured for 24 h at 37 °C prior to infecting mouse mammary glands and mMECs. Selection of *S. uberis* was based on colony characteristics on THA medium, followed by Gram staining, API 20 Strep system (bioMérieux, Lyon, France), Lancefield grouping, and validation by PCR sequencing (Sangon Biotech, Shanghai, China). Isolates were multiplied in Todd–Hewitt broth (THB) liquid medium for 14 h at 37 °C on an orbital shaker at 120 rpm, and stored in 25% glycerol stock solutions at −70 °C. For each challenge, a single loop inoculum of stock solution was added to liquid THB medium containing 2% fetal bovine serum (FBS; Hyclone, Logan, UT, USA) to generate an exponential growth phase (OD600 = 0.5 to 0.7) at 37 °C for infections [32].

### 2.3. Cell Culture and S. uberis Infection

Murine mammary epithelial HC11 cell line (mMECs) (Shanghai Cell Bioscience Inc., Shanghai, China) was used as an in vitro model for infectious mastitis. Cells were cultured in Dulbecco modified Eagle medium (DMEM) supplemented with 10% FBS plus penicillin and streptomycin (100 U/mL each) in T-25/T-75 cell culture flasks. Cells were incubated with 5% CO_2_ at 37 °C and at 80% confluence were harvested by the addition of 2 mL trypsin until 70% cell movement was achieved. Then, cells were collected, centrifuged for 5 min at 1000× *g* and stock solutions prepared in 5% DMSO (dimethyl sulfoxide; Life Tein, LLC, South Plainfield, NJ, USA) for cryopreservation (~1 × 10^8^ cells per cryovial) in liquid nitrogen [32]. Each batch of cells were used from 2–5 passages in a T-25 cell culture flask. For transfection and infection experiments, cells were transferred into 6-well plates (~1 × 10^3^ cells per well) and incubated until the desired confluence. The mMECs were challenged with *S. uberis* at multiplicity of infection (MOI) 5:1, followed by incubation in 5% CO_2_ at 37 °C and subsequently collected for various analyses.

### 2.4. Murine S. uberis Mastitis Model

Mice were housed at the Experimental Animal Center of China Agricultural University with ad libitum access to feed and drinking water and a 12 h light/dark photocycle. Studies were conducted with 20 female albino mice (20 to 23 gm body weight) at 1 wk post parturition and 1 h after separation from their pups. These mice were randomly allocated into five groups (n = 4 mice per group): Sham (Control group); *S. uberis* (*S. uberis* group); *S. uberis* + NAC (*S. uberis* + NAC group); *S. uberis* + Cambinol (*S. uberis* + siSirt1); and *S. uberis* + NK-252 (*S. uberis* + Nrf2 activator). In the *S. uberis* + NAC group, mice were pretreated with an intramammary injection of 300 mg/kg NAC at 3 h prior to *S. uberis* challenge. In *S. uberis* + siSirt1 and Nrf2 activator groups, 50 mg/kg Cambinol and 10 mg/kg NK-252, respectively, were injected 6 h prior to challenge. For *S. uberis* challenge, mice were anesthetized (Zoletil^®^ 50, 0.05 mg/kg IM) and *S. uberis* (1 × 10^5^ CFU/mL diluted in sterile saline, final volume of 100 μL) was injected intramammarily. All intramammary injections were performed with a micro-syringe and 26-gauge needle (using a stereomicroscope to visualize openings) into the 4th nipple (counting forward) of each mouse, after disinfection with 75% alcohol. At 6 h post-inoculation, all mice were euthanized and mammary gland tissues excised.

### 2.5. Determination of ROS Production

An assay kit for ROS (Beyotime Biotechnology, Shanghai, China) was used to measure intracellular ROS concentrations in mMECs seeded in a 6-well plate and challenged with *S. uberis* (MOI 5:1) at 80% confluence. Cells were washed thrice with PBS and incubated in 5% CO_2_ at 37 °C for 30 min in the dark with 10 μM of DCFH-DA fluorescent probe in serum-free DMEM. Then, cells were washed three times with serum-free culture medium, followed by DAPI staining (5 min). Fluorescent signals of stimulated cells were observed with a laser scanning confocal microscope at wavelengths of 405 and 488 nm.

### 2.6. Transmission Electron Microscopy

The mMECs were grown in 6-well plates up to 80% confluence and then challenged by *S. uberis* (MOI 5:1). At 6, 9 and 12 h post infection, cells were digested (500 μL trypsin per well), centrifuged at 1000× *g* for 5 min, washed thrice with PBS and fixed with 2.5% glutaraldehyde for 2 h at room temperature. Cells were stained with 1% osmium tetroxide for 2 h at 4 °C, dried in an ethanol gradient series, and embedded in epoxy resin–acetone mixtures for 2 h. Samples were immersed in resin solution overnight at 37 °C and cut in ultrathin sections (100 nm), and visualized with a light microscope to ensure only a single slice was loaded on a copper grid. Samples were stained with 2% saturated uranyl acetate and treated with 50% ethanol and 3% lead citrate. Copper grid with samples were examined with a transmission electron microscope (TEM; H7650, Tokyo, Japan) at an accelerating voltage of 80 kV.

### 2.7. Western Blot Analysis

The mMECs were cultured in 6-well plates until 80% cell confluence and challenged by *S. uberis* (MOI 5:1). Various pretreatments were used. For example, mMECs were pretreated (1–6 h prior to *S. uberis* infection) with NAC (30 μM) to scavenge intracellular production of ROS, 40 μM Cambinol for Sirt1 inhibition, 10 μM NK-252 for Nrf2 activation or 10 μM ML385 for Nrf2 inhibition.

The mMECs were harvested using a cell scraper on an ice block, rinsed thrice with cold PBS and lysed with RIPA lysis buffer (Beyotime Biotechnology). Supernatants were obtained by centrifugation at 12,000 rpm for 15 min at 4 °C and total protein concentrations determined with a Bicinchoninic acid (BCA) kit (Beyotime Biotechnology).

Protein was loaded into gels (equal amounts in each lane) and β-actin was used as a loading control. After being separated by SDS-PAGE, proteins were transferred to a polyvinylidene difluoride (PVDF) membrane that was washed thrice and blocked with 5% skim milk in tris-buffered saline +0.1% Tween^®^ 20 Detergent (TBST) for 2 h at room temperature. Membranes were incubated overnight at 4 °C with the following primary antibodies: Sirt1 (Cell Signaling, #3931S; Danvers, MA, USA); Keap1 (Abcam, #ab227828; Cambridge, UK); Nrf2 (Proteintech, #16396-1-AP; Rosemont, IL, USA); HO-1 (Proteintech, #27282-1-AP; Rosemont, IL, USA); IL-6 (Invitrogen, #700480; Carlsbad, CA, USA); TNF-α (Proteintech, #17590-1-AP; Rosemont, IL, USA); Atg5 (Proteintech, #10181-2-AP; Rosemont, IL, USA); LC3 (Proteintech, #14600-1-AP; Rosemont, IL, USA); and β-actin (Proteintech #66009-1-Ig; Rosemont, IL, USA). Samples were incubated with secondary antibodies against rabbit or mouse IgG for 2 h at room temperature and signals developed by chemiluminescence using ECL reagents. ImageJ 1.49v software (http://imagej.nih.gov/ij, accessed on 18 December 2023, NIH, Bethesda, MD, USA) was used to assess band density.

### 2.8. T-SOD and MDA Activity

The mMECs were cultured overnight in 6-well plates and treated with NAC 1 h prior to *S. uberis* challenge (MOI 5:1). Total superoxide dismutase (T-SOD) and malondialdehyde (MDA) activity were measured at 6, 12 and 24 h post challenge. The T-SOD activity was measured using a T-SOD assay kit in a microplate reader (Beyotime Biotechnology), whereas MDA activity was evaluated with an MDA ELISA kit (Abcam, Shanghai, China). The same kits were used to determine the activities of T-SOD and MDA in murine mammary glands. Herein, tissues were collected, washed with PBS and stored at −20 °C. Then, tissues were transferred into a mortar with liquid nitrogen, ground to a powder, homogenized with 0.5% Triton X-100 and centrifuged at 14,000× *g* for 5 min at 4 °C to collect supernatants.

### 2.9. Labeling and Tracking Lysosomes

Lysosomes or mature autophagosomes were detected with Lyso-Tracker Red fluorescent probe (Beyotime Biotechnology). Lyso-Tracker Red penetrates mMECs and detects lysosomes or mature autophagosomes in an acidified environment [33]. In these experiments, mMECs were seeded in 6-well plates with coverslips and challenged with *S. uberis* at 80% confluence. Cells were washed thrice with ultra-sterilized PBS and incubated in 5% CO_2_ at 37 °C for 30 min with 50 nM Lyso-Tracker Red in cell culture medium for lysosome red staining. The Lyso-Tracker Red was removed by three washes with PBS and nuclei stained with DAPI for 5 min. Fluorescent signals were observed under a laser scanning confocal microscope at wavelengths of 405, 488 and 561 nm.

### 2.10. Cell Transfection and Confocal Microscopy

Autophagy was monitored using Ad-GFP-LC3 and Ad-mCherry-GFP-LC3B (Beyotime Biotechnology), adenoviruses that express GFP-LC3 and mCherry-GFP-LC3B fusion proteins, respectively [34]. For transfection, mMECs were cultured in 6-well plates with coverslips and incubated for 12 h with 5% CO_2_ at 37 °C. Then, Ad-GFP-LC3B and Ad-mCherry-GFP-LC3B were transfected into cells at 40% confluence in DMEM with 10% FBS, followed by a 24 h incubation. The mMECs were pretreated with Cambinol for Sirt1 inhibition and challenged with *S. uberis* (MOI 5:1) for 12 h. Cells were washed thrice with PBS and cell nuclei were stained with DAPI (10–20 μL per coverslip) for 5 min. After washes with ultra-sterilized PBS, anti-fluorescence quenching sealing solution (8 µL) was added to a clear glass slide. Then, coverslips were inverted on the glass slides and examined with a laser scanning confocal microscope. In an autophagy state, mMECs transfected with Ad-mCherry-GFP-LC3B overexpressed mRFP/mCherry-GFP tandem. Wavelengths of 405, 488 and 561 nm were used for imaging.

### 2.11. Immunofluorescence

The mMECs were cultured in 6-well plates with glass coverslips and incubated overnight at 37 °C with 5% CO_2_ and pretreated (6 h) with a Nrf2 activator or inhibitor before being challenged with *S. uberis* (MOI 5:1) for 6 h. Cells were thrice washed with PBS and fixed with 4% paraformaldehyde for 20 min, followed by permeabilization with 0.25% Triton X-100 in PBS for 15 min. Cells were treated with 3% bovine serum at room temperature for 30 min and incubated with Nrf2 primary antibody overnight at 4 °C. After three PBS washes, cells were incubated with goat anti-rabbit IgG (H + L) tagged with Alexa Fluor 488 for 1 h at room temperature. Cell nuclei were stained with DAPI for 5 min and washed 3 times with PBS. Glass coverslips were inverted on clean glass slides and observed with a laser scanning confocal microscope (Olympus-FV3000, Olympus, Tokyo, Japan) at wavelengths of 405 and 488 nm, with images captured and analyzed with ImageJ software.

### 2.12. Hematoxylin and Eosin Staining of Murine Mammary Gland Tissues

Post *S. uberis* challenge and treatments, mammary gland tissues were excised, dehydrated and fixed in 4% paraformaldehyde for 20 min. Tissues were embedded in paraffin, sectioned (5 μm), treated with xylene for 5 min, and then exposed to 70, 80, 90 and 100% ethanol (10 s in each concentration). Slides were stained with hematoxylin (Beyotime Biotechnology) for 5 min, cleared with tap water for 10 min, rinsed with distilled water and stained with eosin (Beyotime Biotechnology) for 2 min. Slides were washed twice with 70% ethanol. Imaging was performed in a set of randomly chosen fields with an optical microscope (Olympus, Tokyo, Japan) at 100× magnification.

### 2.13. Statistical Analyses

The results of three independent experiments are presented as means ± standard deviation (SD). Data were analyzed by Student’s *t*-test or one-way ANOVA, with Bonferroni correction for multiple comparisons using IBM SPSS statistics 29.0.10 software (https://www.ibm.com; Armonk, NY, USA). *p* < 0.05 and *p* < 0.01 were considered significant and highly significant, respectively.

## 3. Results

### 3.1. S. uberis Enhanced ROS Production and Autophagy Induction in mMECs

**Figure 1:**
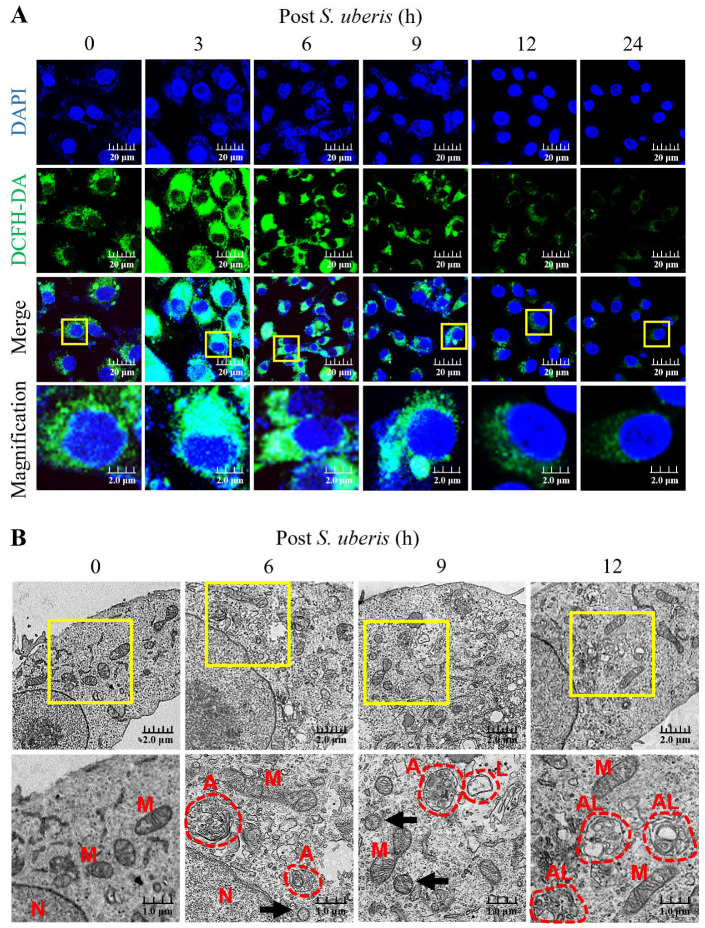
Infecting mMECs with *S. uberis* enhanced intracellular accumulation of ROS and induced autophagy. (**A**) The mMECs were challenged with *S. uberis* and ROS production was determined by measuring the intensity of green fluorescence generated by DCFH-DA and captured with a confocal laser scanning microscope. (**B**) Autophagy induction was confirmed based on morphological and subcellular (TEM) images. Note the nuclei (N), mitochondria (M), autophagosomes (A), lysosomes (L), autolysosome (AL) and *S. uberis* (black arrowheads). Scale bar = 1.0 μm. Magnified figure It comes from the yellow box.

Oxidative stress in *S. uberis*-infected mMECs was gauged by intracellular ROS concentrations until 24 h post *S. uberis* challenge. Dichlorofluorescein diacetate (DCFH-DA) relative fluorescence emissions (as a measure of ROS concentrations) were higher at 3 to 9 h, but decreased by 12 and 24 h post *S. uberis* (Figure 1A). Infection with *S. uberis* induced autophagy and autolysosome formation in mMECs, as observed by transmission electron microscopy (TEM) (Figure 1B). Moreover, infected mMECs had mitochondrial vacuolization, autophagosome and lysosome formation and fusion at 12 h post challenge, whereas *S. uberis* were located intracellularly at 6 and 9 h post challenge.

### 3.2. S. uberis Induced Antioxidative, Inflammatory and Autophagy Markers

**Figure 2:**
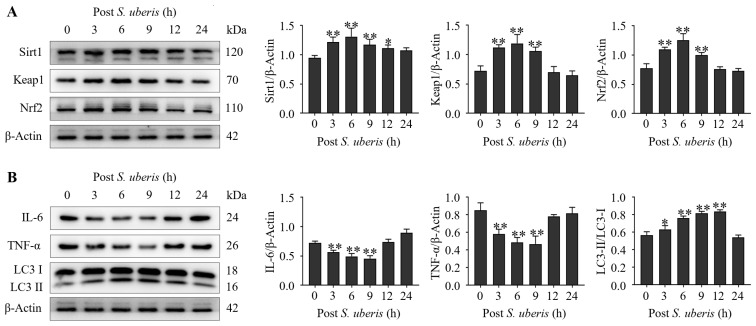
Infecting mMECs with *S. uberis* induced antioxidant and autophagy marker proteins and reduced inflammatory cytokines. (**A**) Western blot analyses of antioxidative Sirt1, Keap1 and Nrf2 isolated from *S. uberis*-challenged mMECs. Proteins were collected up to 24 h post challenge. (**B**) Inflammatory IL-6 and TNF-α, and autophagy of LC3II/LC3I, were also analyzed following *S. uberis* challenge. Mean and standard deviation (three independent experiments) of proteins (quantified with ImageJ 1.49v, http://imagej.nih.gov/ij, accessed on 18 December 2023). * p < 0.05, ** *p* < 0.01 (compared to the Sham).

Expressions of antioxidative Sirt1, Keap1 and Nrf2 were upregulated (*p* < 0.01) in mMECs at 3 to 9 h after *S. uberis* challenge (Figure 2A). Expressions of inflammatory cytokines IL-6 and TNF-α were decreased (*p* < 0.01) at 3 to 9 h, whereas autophagy LC3II/LC3I markers were increased (*p* < 0.01) at 6 to 12 h (Figure 2B). Expression levels of inflammatory cytokines had declined at 3 to 9 hpi (autophagy progression; Supplementary Figure S1).

### 3.3. NAC Pretreatment Mitigated Antioxidative Pathway Elements in S. uberis-Challenged mMECs

**Figure 3:**
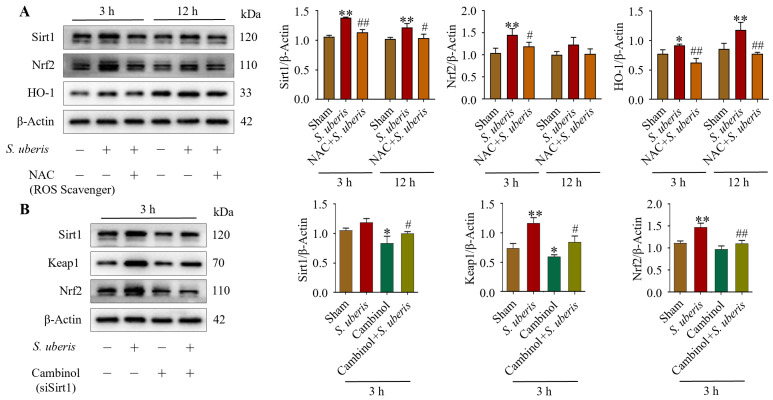
NAC pretreatment in *S. uberis*-challenged mMECs mitigated antioxidative responses. (**A**) Levels of Sirt1, Nrf2 and HO-1 in mMECs pretreated with 30 μM NAC and 1 h later challenged with *S. uberis*. (**B**) Levels of Sirt1, Keap1 and Nrf2 in mMECs pretreated with 40 μM Cambinol (siSirt1) and 6 h later challenged with *S. uberis*. Data were derived from assessments of Western blots and are mean ± SD of three independent trials. * *p* < 0.05 and ** *p* < 0.01 (compared to the Sham); # *p* < 0.05 and ## *p* < 0.01 (compared to the *S. uberis* group).

Antioxidative factors Sirt1, Nrf2 and HO-1 increased at 3 and 12 h post *S. uberis* challenge, but were reduced in NAC-pretreated cells (Figure 3A). Specifically, Sirt1 and Nrf2 expression increased (*p* < 0.01) at 3 h post challenge but it was comparatively lower at 12 h, whereas HO-1 was higher (*p* < 0.01) at 12 versus 3 h post challenge (Supplementary Figure S2). Expressions of Sirt1 were upregulated (not significantly), whereas Keap1 and Nrf2 were upregulated (*p* < 0.01) in *S. uberis*-challenged mMECs at 3 h post challenge; however, these responses were abrogated by Sirt1 inhibition in either transfected cells or those pretreated with Cambinol (Figure 3B). Cambinol/siSirt1 also decreased Keap1 and Nrf2 expression in either the presence or absence of *S. uberis* (Figure 9A).

### 3.4. NAC Treatment Attenuated Inflammation and Cellular Damage in S. uberis-Challenged mMECs/Murine Mammary Glands via Reduced Oxidative Stress

**Figure 4:**
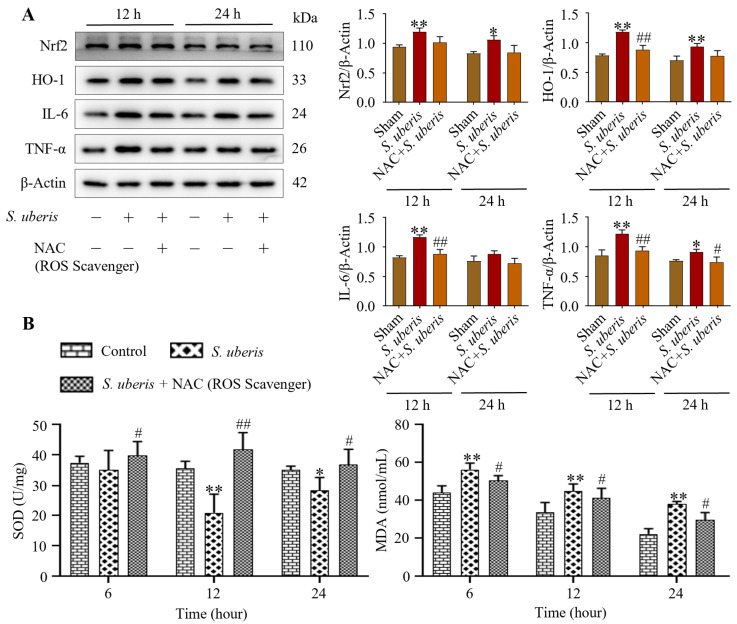
Pretreatment with NAC attenuated inflammatory and cellular damage in *S. uberis*-challenged mMECs and mammary glands by reducing oxidative stress. (**A**) Antioxidative Nrf2 and HO-1 and inflammatory IL-6 and TNF-α were determined in mMECs pretreated with NAC (30 μM) and 1 h later challenged with *S. uberis*. (**B**) Levels of total SOD and MDA activities at 6, 12 and 24 h post *S. uberis* challenge and pretreatment with NAC. Data represent mean ± SD of three independent experiments. * *p* < 0.05 and ** *p* < 0.01 (compared to the Sham); # *p* < 0.05 and ## *p* < 0.01 (compared to the *S. uberis* group).

*S. uberis*-challenged mMECs pretreated with NAC showed reduced oxidative stress. Although Nrf2, HO-1, IL-6 and TNF-α expression increased (*p* < 0.01) at 12 and 24 h post *S. uberis* challenge (Figure 4A), these levels were lesser in mMECs pretreated with NAC (Supplementary Figure S3). Activity of T-SOD in the *S. uberis*-challenged group was lower at 12 h (*p* < 0.01) and 24 h (*p* < 0.05) than at 6 h post *S. uberis* challenge (Figure 4B). Total SOD increased (*p* < 0.05) in the NAC + *S. uberis* group compared to the *S. uberis* group, but MDA activity was higher (*p* < 0.05) at 6 h post *S. uberis* compared to 12 or 24 h (Figure 4). In cells pretreated with NAC + *S. uberis*, MDA activity was progressively reduced at 6, 12 and 24 h.

### 3.5. NAC Treatment Attenuated Autophagy Mechanism in S. uberis-Challenged mMECs

**Figure 5:**
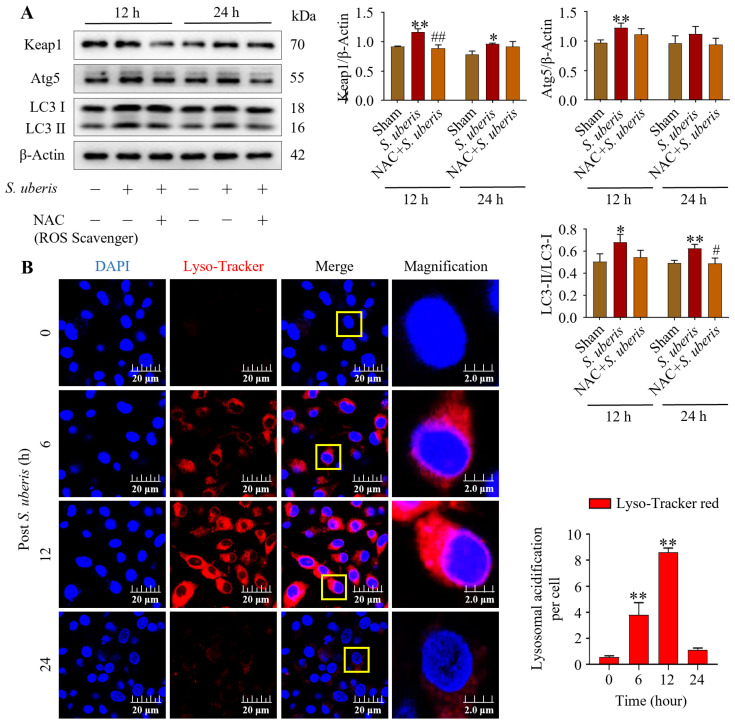
NAC treatment alleviated autophagy in *S. uberis*-challenged mMECs. (**A**) Protein concentrations of Keap1, Atg5 and LC3II/LC3I in mMECs pretreated with NAC (30 μM) 1 h prior to *S. uberis* challenge. (**B**) Formation of acidified lysosomes was quantified by measuring red fluorescence intensity. In total, 30 cells were selected for each group and 10 cells per sample with three repeats were quantified for statistical analyses. Data are representative means of three independent experiments. * *p* < 0.05 and ** *p* < 0.01 (compared to the Sham); # *p* < 0.05 and ## *p* < 0.01 (compared to the *S. uberis* group). Magnified figure It comes from the yellow box.

Pretreatment with NAC in the presence of *S. uberis* impaired autophagy and antioxidative Keap1 at 12 and 24 h post challenge. Levels of Keap1, Atg5 and LC3II/LC3I were upregulated (*p* < 0.01) in *S. uberis*-challenged groups (Figure 5A); these levels peaked at 12 h compared to 24 h post *S. uberis* in cells pretreated with NAC (Supplementary Figure S4). Increased Lyso-Tracker red fluorescence (*p* < 0.01), indicative of autophagy, was observed in *S. uberis*-challenged mMECs (Figure 5B). Red fluorescence intensity increased from 0 to 6 and 12 h post challenge, but had subsequently decreased by 24 h.

### 3.6. Inhibition of Antioxidative Sirt1 Impaired Autophagy in mMECs by Inducing Inflammation

**Figure 6:**
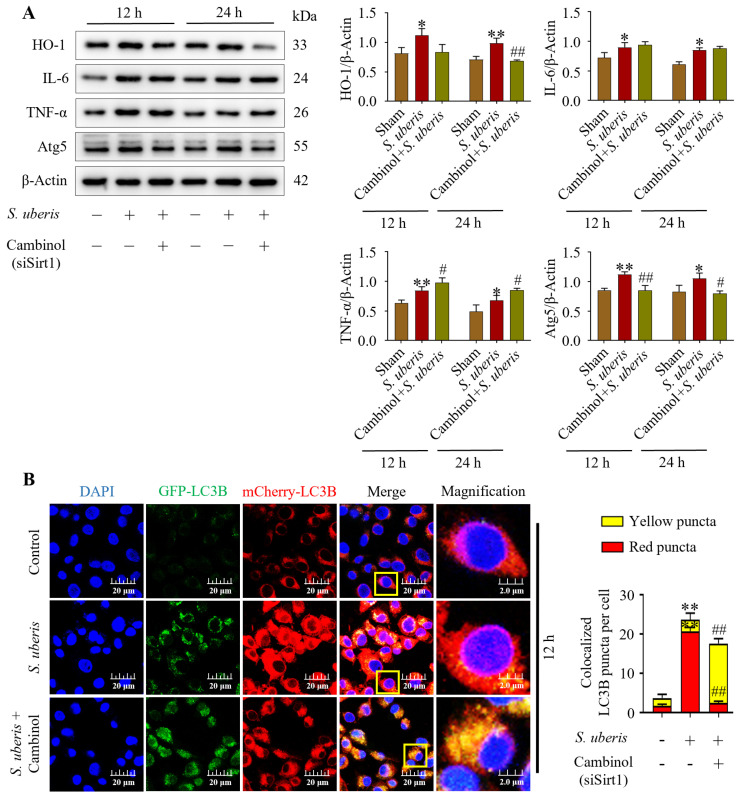
Silencing of Sirt1 in mMECs inhibited autophagy by inducing inflammation. (**A**) Protein expressions of HO-1, IL-6, TNF-α and Atg5 in mMECs pretreated with Cambinol (siSirt1) (40 μM) 6 h prior to *S. uberis* challenge. (**B**) Transfection of mMECs with Ad-mCherry-GFP-LC3B to determine autophagy inhibition in Cambinol treatment. Quantification of red, green and yellow fluorescence conducted by ImageJ 1.49v software with JaCoP plugin. Statistical data analyses were conducted on 10 cells per sample. Data represent mean ± SD of three independent experiments. * *p* < 0.05 and ** *p* < 0.01 (compared to the Sham); # *p* < 0.05 and ## *p* < 0.01 (compared to the *S. uberis* group). Magnified figure It comes from the yellow box.

Concentrations of HO-1, IL-6, TNF-α and Atg5 were upregulated (*p* < 0.05) in mMECs challenged by *S. uberis*, but concentrations were reduced in mMECs pretreated with Sirt1 inhibitor (Cambinol) before *S. uberis* challenge (Figure 6A). In response to siSirt1 treatment, expressions of IL-6 increased numerically along with increases (*p* < 0.05) in TNF-α markers (Supplementary Figure S5). Autophagy dynamics changed in response to Cambinol. Vector Ad-mCherry-GFP-LC3B co-localized in autophagosomes in *S. uberis*-challenged cells (Figure 6B), but in Cambinol + *S. uberis* cells, the red fluorescence turned into yellow puncta (*p* < 0.01) due to a green GFP-LC3B co-localization in a non-acidic environment, indicative of autophagy inhibition.

### 3.7. Antioxidative Nrf2 Activation Induced Autophagy by Alleviating Inflammatory Responses

**Figure 7:**
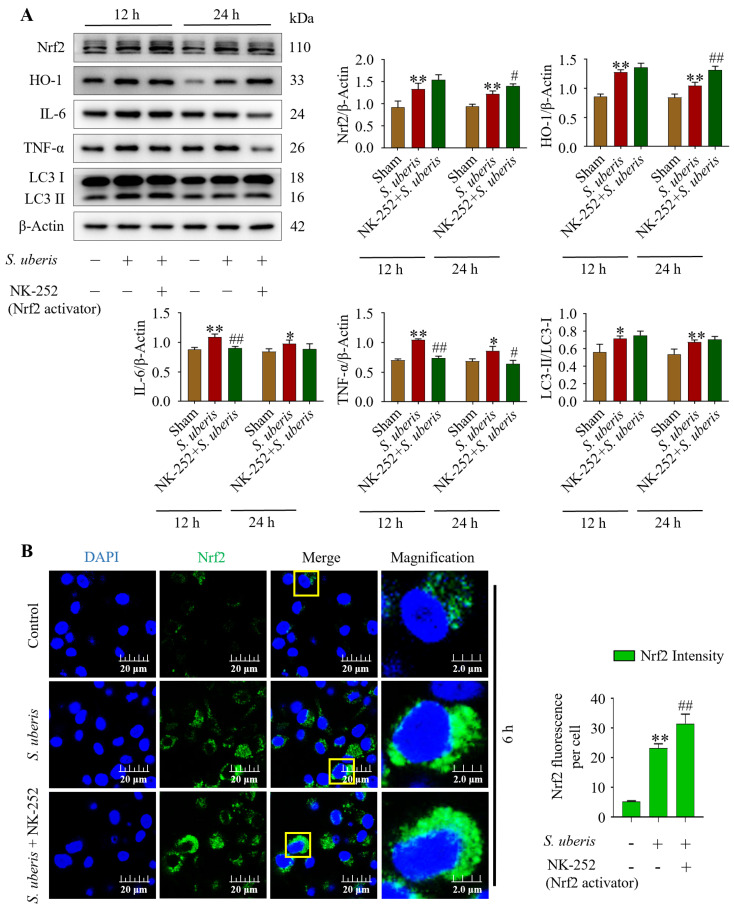
Activation of Nrf2 triggered autophagy by attenuating IL-6 and TNF-α. (**A**) Expression of Nrf2, HO-1, IL-6, TNF-α and LC3II/LC3I in mMECs pretreated with 10 μM of NK-252 (Nrf2 activator) 6 h prior to *S. uberis* challenge. (**B**) Nrf2 protein expressions in *S. uberis*-challenged mMECs analyzed by immunofluorescence assay. Nrf2 green fluorescence intensity was quantified using ImageJ software with JaCoP plugin. For statistical analyses, 10 cells were selected for each sample. The given data are representative of the mean of three independent experiments. * *p* < 0.05 and ** *p* < 0.01 (compared to the Sham); # *p* < 0.05 and ## *p* < 0.01 (compared to the *S. uberis* group). Magnified figure It comes from the yellow box.

At 12 and 24 hpi, concentrations of antioxidants Nrf2, HO-1, inflammatory IL-6, TNF-α and autophagy LC3II/LC3I markers were evaluated in the presence of NK-252 (Nrf2 activator) to identify the effects of the antioxidative pathway on inflammation and autophagy mechanisms. The mMECs infected with *S. uberis* expressed increased (*p* < 0.01) concentrations of Nrf2, HO-1, IL-6, and TNF-α and increased LC3II/LC3I markers compared to the Sham (Figure 7A). However, IL-6 and TNF-α had decreased (*p* < 0.01) at 12 hpi with NK-252 + infection compared to infection of *S. uberis* (Supplementary Figure S6). Additionally, green fluorescence in the *S. uberis* + NK-252 group had stronger intensity (*p* < 0.01) than mMECs infected with *S. uberis* or non-infected mMECs (Sham) (Figure 7B), indicating Nrf2 activation at 6 hpi.

### 3.8. Deactivation of Antioxidative Nrf2 Inhibited Autophagy by Increasing Inflammatory Responses

**Figure 8:**
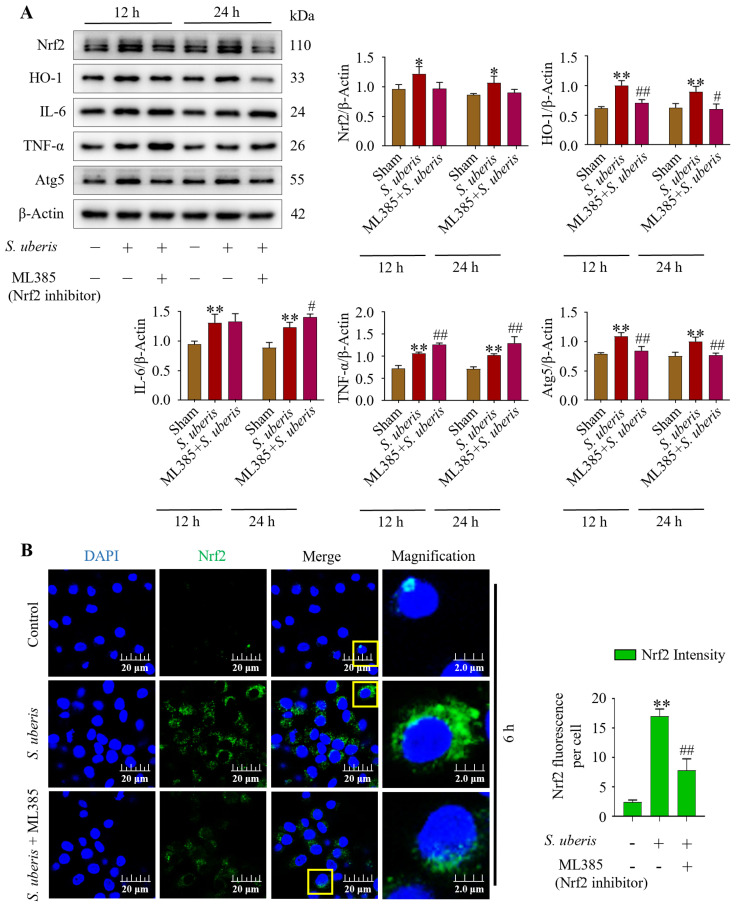
Suppression of antioxidative Nrf2 inhibited autophagy mechanism by increasing inflammation. (**A**) Nrf2 marker proteins were silenced by using 10 μM of ML385 in mMECs and Nrf2, HO-1, IL-6, TNF-α and Atg5 protein expression was analyzed at 12 and 24 h post *S. uberis* challenge. (**B**) Immunofluorescence confocal assay of Nrf2 in *S. uberis*-challenged mMECs. Quantitative image analysis of green Nrf2 intensity was conducted by ImageJ with JaCoP plugin in 10 cells/sample. Represented data are mean ± SD of three independent trials. * *p* < 0.05 and ** *p* < 0.01 (compared to the Sham); # *p* < 0.05 and ## *p* < 0.01 (compared to the *S. uberis* group). Magnified figure It comes from the yellow box.

Infection with *S. uberis* induced (*p* < 0.01) Nrf2, HO-1, IL-6, TNF-α and Atg5 (Figure 8A). However, pretreatment with ML385 (Nrf2 inhibitor) followed by *S. uberis* challenge numerically decreased Nrf2, whereas HO-1 and Atg5 were reduced (*p* < 0.01) (Supplementary Figure S7). Concentrations of IL-6 increased (*p* > 0.05) and TNF-α was upregulated (*p* < 0.01) after Nrf2 inhibition, whereas HO-1 and autophagy Atg5 marker were downregulated (*p* < 0.01). Deactivation of Nrf2 in the presence of *S. uberis* was evident based on lower green fluorescence intensity (*p* < 0.01) in the ML385 + *S. uberis* group versus the *S. uberis* group (Figure 8B).

### 3.9. Antioxidant Factors Were Increased in S. uberis-Challenged mMECs and Mammary Glands

**Figure 9:**
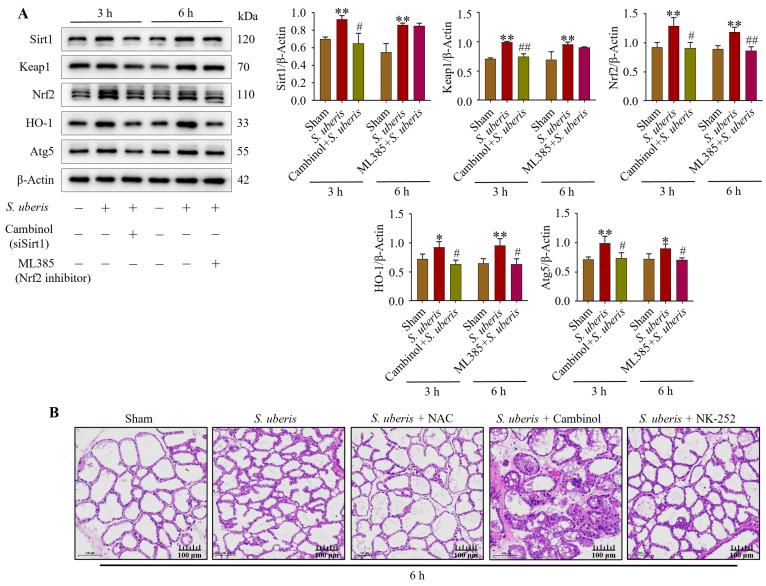
Mammary glands and mMECs activated antioxidative factors in response to oxidative stress provoked by *S. uberis*. (**A**) Expression of antioxidants Sirt1, Keap1, Nrf2, HO-1 and Atg5 in mMECs silenced in Sirt1 and Nrf2 previous to *S. uberis* at 3 h and 6 h challenge. (**B**) Mammary gland morphology after *S. uberis* challenge for 6 h in mice pretreated with NAC, Cambinol or NK-252. * *p* < 0.05 and ** *p* < 0.01 (compared to the Sham); # *p* < 0.05 and ## *p* < 0.01 (compared to the *S. uberis* group).

Expression of Sirt1, Keap1, Nrf2, HO-1 and Atg5 was upregulated (*p* < 0.01) in *S. uberis*-challenged mMECs (Figure 9A). However, silencing of Sirt1 with Cambinol downregulated (*p* < 0.05) Sirt1, Keap1, Nrf2, HO-1 and Atg5 at 3 h, whereas inhibition of Nrf2 downregulated (*p* < 0.05) Nrf2, HO-1 and Atg5 at 6 h (Supplementary Figure S8). Therefore, Sirt1 and Keap1 preceded Nrf2 and HO-1 in the antioxidative pathway. In the murine model of mastitis, Sham mammary glands did not have sloughed mammary epithelial cells (Figure 9B). In contrast, mammary glands infected with *S. uberis* had damaged mammary epithelial cells, irrespective of Cambinol treatment. Compared to the *S. uberis* and Cambinol + *S. uberis* group, histopathological inflammatory changes were lower in the NAC + *S. uberis* and NK-252 + *S. uberis* groups.

## 4. Discussion

As an important mediator of inflammation, *Streptococcus uberis* has been widely used to establish mastitis models in mice [35]. The mouse mastitis model established by injection of *S. uberis* was consistent with the clinical symptoms of cow mastitis [36]. The inflammatory factors TNF-α, IL-6 and IL-1β are closely associated with the response to bacterial *S. uberis* infection. *S. uberis* can stimulate inflammation cytokines, including IL-1β, TNF-α, IL-6 and IL-8, in bovine neutrophils, mammary epithelial cell and somatic milk cells models [37,38]. *S. uberis* is an important promoter in the NF-κB and MAPK inflammatory signaling pathways, which triggers inflammation in mammary epithelial cells [35]. Both TLR2 and TLR4 are involved in sensing *S. uberis* invasion. TLR2 is a principal receptor. The ability to stimulate TLR2 expression on the cell surface resulted in significant upregulation of both NF-κB and MAPK signaling pathways downstream [39]. In this study, murine mastitis models and mMECs were infected with *S. uberis* and the antioxidative, inflammatory and autophagy pathway elements were evaluated. We tested the hypothesis that silencing/activation of antioxidative pathway factors will bring changes in the autophagy mechanism. Whereas inflammatory changes and autophagy mechanisms were inversely correlated in our recent study [32], highlighting the role of Nrf2 in alleviating inflammation [40], this study examined the involvement of antioxidative pathways regulating autophagy. Key antioxidative elements, including Sirt1, Keap1, Nrf2 and HO-1 positively modulated the autophagy markers Atg5 and LC3II/LC3I and concurrently downregulated inflammatory IL-6 and TNF-α. Antioxidant SOD concentrations decreased, along with a reduction in MDA activity [41]. Thus, lower T-SOD and MDA activities reduced cellular damage due to oxidative stress in *S. uberis*-infected mammary glands, particularly epithelial cells. Furthermore, treatment with NAC attenuated morphological damage and reduced expressions of inflammatory markers but not autophagy, implying that oxidative stress and activation of the antioxidative pathway are key for inducing autophagy.

In response to oxidative stress at early stages of *S. uberis* infection, the antioxidative pathway was activated with increased key antioxidative regulatory factors (Sirt1, Keap1, Nrf2 and HO-1) [14,42]. Expression of inflammatory and autophagy markers also fluctuated with *S. uberis* infection. Therefore, the antioxidative pathway seemed to induce autophagy by modulating inflammatory responses. As inflammation and oxidative stress are directly associated, and excessive ROS activates numerous inflammatory mechanisms [43], we evaluated pathways associated with oxidative stress and mastitis. Inhibition of ROS with NAC prior to *S. uberis* infection reduced the need for antioxidative factors in mammary glands and mMECs. Furthermore, lower T-SOD and MDA activities also reduced oxidative stress damage. Thus, inflammatory IL-6 and TNF-α were downregulated, whereas autophagy Atg5 and LC3II/LC3I and the antioxidative Sirt1, Keap1, Nrf2 and HO-1 were increased.

In the present study, the antioxidative pathway was induced by *S. uberis* and counteracted oxidative stress in both mMECs and mastitis models. Sirt1 is the key element, as it reduced oxidative stress, whereas increases in Keap1, Nrf2 and HO-1 were dependent on Sirt1 marker proteins (indicating enhanced antioxidative activity) and suppressing inflammation. Nrf2 is a crucial regulator of cellular redox reactions [44] and a sensitive signal for scavenging ROS. Some pathogenic microbes enhance oxidative stress associated with expressions of the Nrf2/HO-1 pathway [45]. Similar outcomes following treatment with ML385 (Nrf2 inhibitor) and Cambinol (siSirt1) after *S. uberis* challenge implied that both Sirt1 and Nrf2 are antioxidative markers. Lack of suppression of Sirt1 and Keap1 with ML385 (Nrf2 inhibitor) indicated that Nrf2 and HO-1 are later components and led by Sirt1 and Keap1 antioxidative elements. Increases in the cytoprotective gene HO-1 after activation of Nrf2 would protect mMECs against oxidative damage [46], degrading heme to generate anti-inflammatory carbon monoxide (a vasodilatory gas) [47]. Moreover, downregulation of IL-6 and TNF-α with increased HO-1 due to activation of Nrf2 would promote autophagy.

Activation of the antioxidative pathway would stimulate autophagy by decreasing inflammation. IL-6 and TNF-α were downregulated during autophagy [32], along with increased expressions of antioxidative Sirt1, Keap1, Nrf2 and HO-1. Thus, inhibition of Nrf2 not only reduced antioxidant activity but also upregulated inflammatory IL-6 and TNF-α markers. Additionally, the antioxidative pathway had a positive and direct impact on autophagy. In fact, silencing Sirt1 impaired autophagy in the Ad-mCherry-GFP-LC3B transfection assay, with both GFP green and mCherry red fluorescence becoming yellow puncta [48].

We illustrated that *S. uberis* challenge indirectly induces autophagy in mMECs by targeting antioxidative Sirt1, Keap1, Nrf2 and Ho-1. Oxidative stress was increased, which also increased Sirt1 along with Keap1, Nrf2 and HO-1 as antioxidative mechanisms. Oxidative stress also dissociates Nrf2 from Keap1 protein, transfers Nrf2 to the nucleus, and induced expressions of HO-1. The increased antioxidative factors increase autophagous Atg5 and LC3II/LC3I by reducing inflammatory IL-6 and TNF-α. The inhibitory treatment of Sirt1 and Nrf2 have negative effects on autophagy induction, while Nrf2 activation enhanced autophagy activity. HO-1 increased the levels of total SOD and MDA contents against oxidative stress. Thus, autophagy inhibits oxidative stress and cellular damage in mastitis.

## 5. Conclusions

**Figure 10:**
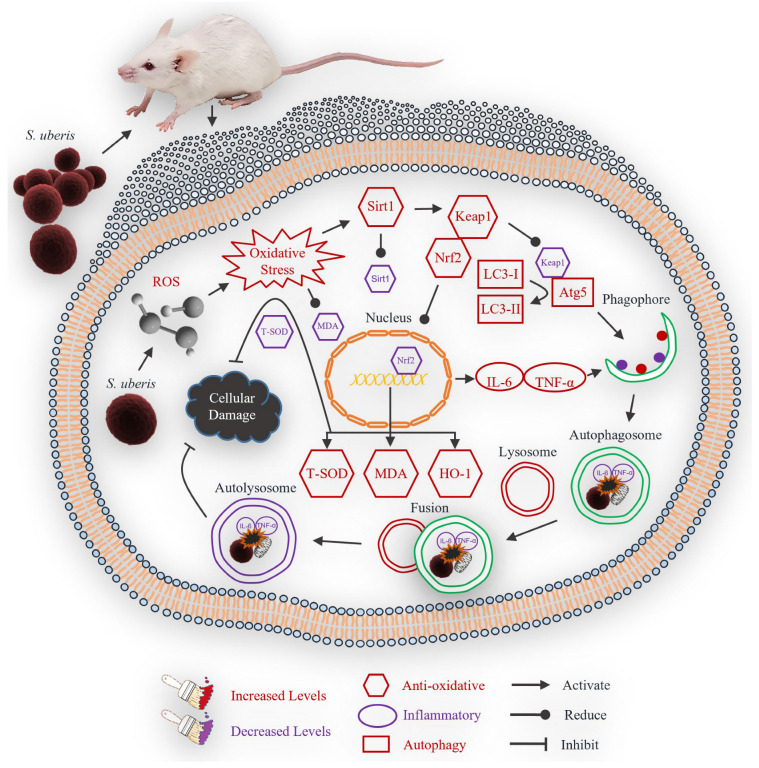
Theoretical scheme of antioxidative factors that regulate autophagy mechanism in mastitis.

In conclusion, *S. uberis* infection induced oxidative stress in mouse mammary epithelial cells, which activated an antioxidative pathway that mitigated inflammatory responses and reduced cellular damage (Figure 10). Inflammation could thus be attenuated by the antioxidative pathway and autophagy mechanism.

## Data Availability

Data will be made available on request from the corresponding author.

## Supplementary Materials

The following supporting information can be downloaded at: https://www.mdpi.com/article/10.3390/antiox13020171/s1. Figure S1: Original WB of *S. uberis*-infected mMECs induced antioxidant and autophagy marker proteins and reduced inflammatory cytokines; Figure S2: Original WB of NAC pretreatment in *S. uberis*-challenged mMECs mitigated antioxidative responses; Figure S3: Original WB of pretreatment with NAC attenuated inflammatory and cellular damage in *S. uberis*-challenged mMECs and mammary glands by reducing oxidative stress; Figure S4: Original WB of NAC treatment alleviated autophagy in *S. uberis*-challenged mMECs; Figure S5: Original WB of silencing of Sirt1 in mMECs inhibited autophagy by inducing inflammation; Figure S6: Original WB of activation of Nrf2 triggered autophagy by attenuating IL-6 and TNF-α; Figure S7: Original WB of suppression of antioxidative Nrf2 inhibited autophagy mechanism by increasing inflammation; Figure S8: Original WB of mammary glands and mMECs activated antioxidative factors in response to oxidative stress provoked by *S. uberis*.

## References

[1] Majumder S., Eckersall P.D., George S. (2023). Bovine mastitis: Examining factors contributing to treatment failure and prospects of nano-enabled antibacterial combination therapy. ACS Agric. Sci. Technol..

[2] Dalanezi F., Joaquim S., Guimarães F., Guerra S., Lopes B., Schmidt E., Cerri R., Langoni H. (2020). Influence of pathogens causing clinical mastitis on reproductive variables of dairy cows. J. Dairy Sci..

[3] Heikkilä A.M., Liski E., Pyörälä S., Taponen S. (2018). Pathogen-specific production losses in bovine mastitis. J. Dairy Sci..

[4] Wente N., Klocke D., Paduch J.H., Zhang Y., Tho Seeth M., Zoche-Golob V., Reinecke F., Mohr E., Krömker V. (2019). Associations between *Streptococcus uberis* strains from the animal environment and clinical bovine mastitis cases. J. Dairy Sci..

[5] de Jong E., McCubbin K.D., Speksnijder D., Dufour S., Middleton J.R., Ruegg P.L., Lam T.J.G.M., Kelton D.F. (2023). Invited review: Selective treatment of clinical mastitis in dairy cattle. J. Dairy Sci..

[6] Wang Z., Lan R., Xu Y., Zuo J., Han X., Phouthapane V., Luo Z., Miao J. (2021). Taurine alleviates *Streptococcus uberis*-induced inflammation by activating autophagy in mammary epithelial cells. Front. Immunol..

[7] Zhou Y., Lan R., Xu Y., Zhou Y., Lin X., Miao J. (2020). Resveratrol alleviates oxidative stress caused by *Streptococcus uberis* infection via activating the Nrf2 signaling pathway. Int. Immunopharmacol..

[8] Hu W., Chan H., Lu L., Wong K.T., Wong S.H., Li M.X., Xiao Z.G., Cho C.H., Gin T., Chan M.T.V. (2020). Autophagy in intracellular bacterial infection. Semin. Cell Dev. Biol..

[9] Yu L., Chen Y., Tooze S.A. (2018). Autophagy pathway: Cellular and molecular mechanisms. Autophagy.

[10] Romeo M.A., Montani M.S.G., Benedetti R., Giambelli L., D’Aprile R., Gaeta A., Faggioni A., Cirone M. (2021). The cross-talk between STAT1/STAT3 and ROS up-regulates PD-L1 and promotes the release of pro-inflammatory/immune suppressive cytokines in primary monocytes infected by HHV-6B. Virus Res..

[11] Ren Z., He H., Zuo Z., Xu Z., Wei Z., Deng J. (2019). The role of different SIRT1-mediated signaling pathways in toxic injury. Cell. Mol. Biol. Lett..

[12] Alshehri A., El-Kott A.F., Eleawa S., El-Gerbed M., Khalifa H., El-Kenawy A., Albadrani G.M., Abdel-Daim M.M. (2021). Kaempferol protects against streptozotocin-induced diabetic cardiomyopathy in rats by a hypoglycemic effect and upregulating SIRT1. J. Physiol. Pharmacol..

[13] Zhao H., Chen S., Gao K., Zhou Z., Wang C., Shen Z., Guo Y., Li Z., Wan Z., Liu C. (2017). Resveratrol protects against spinal cord injury by activating autophagy and inhibiting apoptosis mediated by the SIRT1/AMPK signaling pathway. Neuroscience.

[14] Huang K., Gao X., Wei W. (2017). The crosstalk between Sirt1 and Keap1/Nrf2/ARE anti-oxidative pathway forms a positive feedback loop to inhibit FN and TGF-β1 expressions in rat glomerular mesangial cells. Exp. Cell Res..

[15] Baird L., Yamamoto M. (2020). The molecular mechanisms regulating the KEAP1-NRF2 pathway. Mol. Cell Biol..

[16] Mizunoe Y., Kobayashi M., Sudo Y., Watanabe S., Yasukawa H., Natori D., Hoshino A., Negishi A., Okita N., Komatsu M. (2018). Trehalose protects against oxidative stress by regulating the Keap1–Nrf2 and autophagy pathways. Redox Biol..

[17] Shen K., Jia Y., Wang X., Zhang J., Liu K., Wang J., Cai W., Li J., Li S., Zhao M. (2021). Exosomes from adipose-derived stem cells alleviate the inflammation and oxidative stress via regulating Nrf2/HO-1 axis in macrophages. Free Radic. Biol. Med..

[18] Chen H., Xie K., Chen Y., Wang Y., Wang Y., Lian N., Zhang K., Yu Y. (2019). Nrf2/HO-1 signaling pathway participated in the protection of hydrogen sulfide on neuropathic pain in rats. Int. Immunopharmacol..

[19] Jayasinghe A.M.K., Kirindage K.G.I.S., Fernando I.P.S., Han E.J., Oh G.W., Jung W.K., Ahn G. (2022). Fucoidan isolated from sargassum confusum suppresses inflammatory responses and oxidative stress in TNF-α/IFN-γ-stimulated HaCaT keratinocytes by activating Nrf2/HO-1 signaling pathway. Mar. Drugs.

[20] Shen C., Luo Z., Ma S., Yu C., Gao Q., Zhang M., Zhang H., Zhang J., Xu W., Yao J. (2022). Microbe-derived antioxidants reduce lipopolysaccharide-induced inflammatory responses by activating the Nrf2 pathway to inhibit the ROS/NLRP3/IL-1β signaling pathway. Int. J. Mol. Sci..

[21] Ichimura Y., Komatsu M. (2018). Activation of p62/SQSTM1–Keap1–nuclear factor erythroid 2-related factor 2 pathway in cancer. Front. Oncol..

[22] Fan X., Huang T., Tong Y., Fan Z., Yang Z., Yang D., Mao X., Yang M. (2022). p62 works as a hub modulation in the ageing process. Ageing Res. Rev..

[23] Debnath J., Gammoh N., Ryan K.M. (2023). Autophagy and autophagy-related pathways in cancer. Nat. Rev. Mol. Cell Biol..

[24] Xu W., Ocak U., Gao L., Tu S., Lenahan C.J., Zhang J., Shao A. (2021). Selective autophagy as a therapeutic target for neurological diseases. Cell. Mol. Life Sci..

[25] Ding X., Zhu C., Wang W., Li M., Ma C., Gao B. (2023). SIRT1 is a regulator of autophagy: Implications for the progression and treatment of myocardial ischemia-reperfusion. Pharmacol. Res..

[26] Sudhakar P., Jacomin A.C., Hautefort I., Samavedam S., Fatemian K., Ari E., Gul L., Demeter A., Jones E., Korcsmaros T. (2019). Targeted interplay between bacterial pathogens and host autophagy. Autophagy.

[27] King K.E., Losier T.T., Russell R.C. (2021). Regulation of autophagy enzymes by nutrient signaling. Trends Biochem. Sci..

[28] Galluzzi L., Bravo-San Pedro J.M., Levine B., Green D.R., Kroemer G. (2017). Pharmacological modulation of autophagy: Therapeutic potential and persisting obstacles. Nat. Rev. Drug Discov..

[29] Zhao W., Xu M., Barkema H.W., Xie X., Lin Y., Khan S., Kastelic J.P., Wang D., Deng Z., Han B. (2022). *Prototheca bovis* induces autophagy in bovine mammary epithelial cells via the HIF-1α and AMPKα/ULK1 pathway. Front. Immunol..

[30] Xu M., Liu Y., Mayinuer T., Lin Y., Wang Y., Gao J., Wang D., Kastelic J.P., Han B. (2022). *Mycoplasma bovis* inhibits autophagy in bovine mammary epithelial cells via a PTEN/PI3K-Akt-mTOR-dependent pathway. Front. Microbiol..

[31] Liu Y., Deng Z., Xu S., Liu G., Lin Y., Khan S., Gao J., Qu W., Kastelic J.P., Han B. (2021). *Mycoplasma bovis* subverts autophagy to promote intracellular replication in bovine mammary epithelial cells cultured in vitro. Vet. Res..

[32] Khan S., Yang J., Cobo E.R., Wang Y., Xu M., Wang T., Shi Y., Liu G., Han B. (2023). *Streptococcus uberis* induced expressions of pro-inflammatory IL-6, TNF-α, and IFN-γ in bovine mammary epithelial cells associated with inhibited autophagy and autophagy flux formation. Microb. Pathog..

[33] Ruan L., Bai J., Ji X., Zhao W., Dong X. (2022). A series of meso amide BODIPY based lysosome-targeting fluorescent probe with high photostability and sensitivity. Anal. Chim. Acta.

[34] Klionsky D.J., Abdel-Aziz A.K., Abdelfatah S., Abdellatif M., Abdoli A., Abel S., Abel S., Abeliovich H., Abildgaard M.H., Abudu Y.P. (2021). Guidelines for the use and interpretation of assays for monitoring autophagy. Autophagy.

[35] Zhou G., Zhang W., Wen H., Su Q., Hao Z., Liu J., Gao Y., Zhang H., Ge B., Tong C. (2023). Esculetin improves murine mastitis induced by *Streptococcus* isolated from bovine mammary glands by inhibiting NF-κB and MAPK signaling pathways. Microb. Pathog..

[36] Vander Elst N., Bellemans J., Lavigne R., Briers Y., Meyer E. (2024). Endolysin NC5 improves early cloxacillin treatment in a mouse model of *Streptococcus uberis* mastitis. Appl. Microbiol. Biotechnol..

[37] Goto S., Mikami O., Nagasawa Y., Watanabe A. (2023). Bovine neutrophils stimulated with *Streptococcus uberis* induce neutrophil extracellular traps and cause cytotoxicity, and transcriptional upregulation of inflammatory cytokine genes in bovine mammary epithelial cells. J. Vet. Med. Sci..

[38] Wellnitz O., Berger U., Schaeren W., Bruckmaier R. (2012). Mastitis severity induced by two Streptococcus uberis strains is reflected by the mammary immune response in vitro. Schweiz Arch. Tierheilkd..

[39] Li B., Xi P., Wang Z., Han X., Xu Y., Zhang Y., Miao J. (2018). PI3K/Akt/mTOR signaling pathway participates in *Streptococcus uberis*-induced inflammation in mammary epithelial cells in concert with the classical TLRs/NF-ĸB pathway. Vet. Microbiol..

[40] Zhao W., Deng Z., Barkema H.W., Xu M., Gao J., Liu G., Lin Y., Kastelic J.P., Han B. (2022). Nrf2 and NF-κB/NLRP3 inflammasome pathways are involved in *Prototheca bovis* infections of mouse mammary gland tissue and mammary epithelial cells. Free Radic. Biol. Med..

[41] Zalewska-Ziob M., Adamek B., Kasperczyk J., Romuk E., Hudziec E., Chwalińska E., Dobija-Kubica K., Rogoziński P., Bruliński K. (2019). Activity of antioxidant enzymes in the tumor and adjacent noncancerous tissues of non-small-cell lung cancer. Oxid. Med. Cell Longev..

[42] Pu Q., Guo X.X., Hu J.J., Li A.L., Li G.G., Li X.Y. (2022). Nicotinamide mononucleotide increases cell viability and restores tight junctions in high-glucose-treated human corneal epithelial cells via the SIRT1/Nrf2/HO-1 pathway. Biomed. Pharmacother..

[43] Zhao M., Wang Y., Li L., Liu S., Wang C., Yuan Y., Yang G., Chen Y., Cheng J., Lu Y. (2021). Mitochondrial ROS promote mitochondrial dysfunction and inflammation in ischemic acute kidney injury by disrupting TFAM-mediated mtDNA maintenance. Theranostics.

[44] Wei W., Ma N., Fan X., Yu Q., Ci X. (2020). The role of Nrf2 in acute kidney injury: Novel molecular mechanisms and therapeutic approaches. Free Radic. Biol. Med..

[45] Molagoda I.M.N., Lee K.T., Choi Y.H., Kim G.Y. (2020). Anthocyanins from Hibiscus syriacus L. inhibit oxidative stress-mediated apoptosis by activating the Nrf2/HO-1 signaling pathway. Antioxidants.

[46] Xu D., Liu J., Ma H., Guo W., Wang J., Kan X., Li Y., Gong Q., Cao Y., Cheng J. (2020). Schisandrin A protects against lipopolysaccharide-induced mastitis through activating Nrf2 signaling pathway and inducing autophagy. Int. Immunopharmacol..

[47] Petsouki E., Cabrera S.N.S., Heiss E.H. (2022). AMPK and NRF2: Interactive players in the same team for cellular homeostasis?. Free Radic. Biol. Med..

[48] Ma J., Han Z., Jiao R., Yuan G., Ma C., Yan X., Meng A. (2023). Irisin ameliorates PM2. 5-induced acute lung injury by regulation of autophagy through AMPK/mTOR pathway. J. Inflamm. Res..

