# Supplementary material for: Antioxidative Sirt1 and the Keap1-Nrf2 Signaling Pathway Impair Inflammation and Positively Regulate Autophagy in Murine Mammary Epithelial Cells or Mammary Glands Infected with Streptococcus uberis
Source: Antioxidants (Basel). 2024 Jan 29;13(2):171. doi: 10.3390/antiox13020171 (PMC10886112; doi:10.3390/antiox13020171)
Supplement: Supplementary file 1 [file antioxidants-13-00171-s001.zip › antioxidants-2806628-supplementary.pdf]

Supplementary Data:

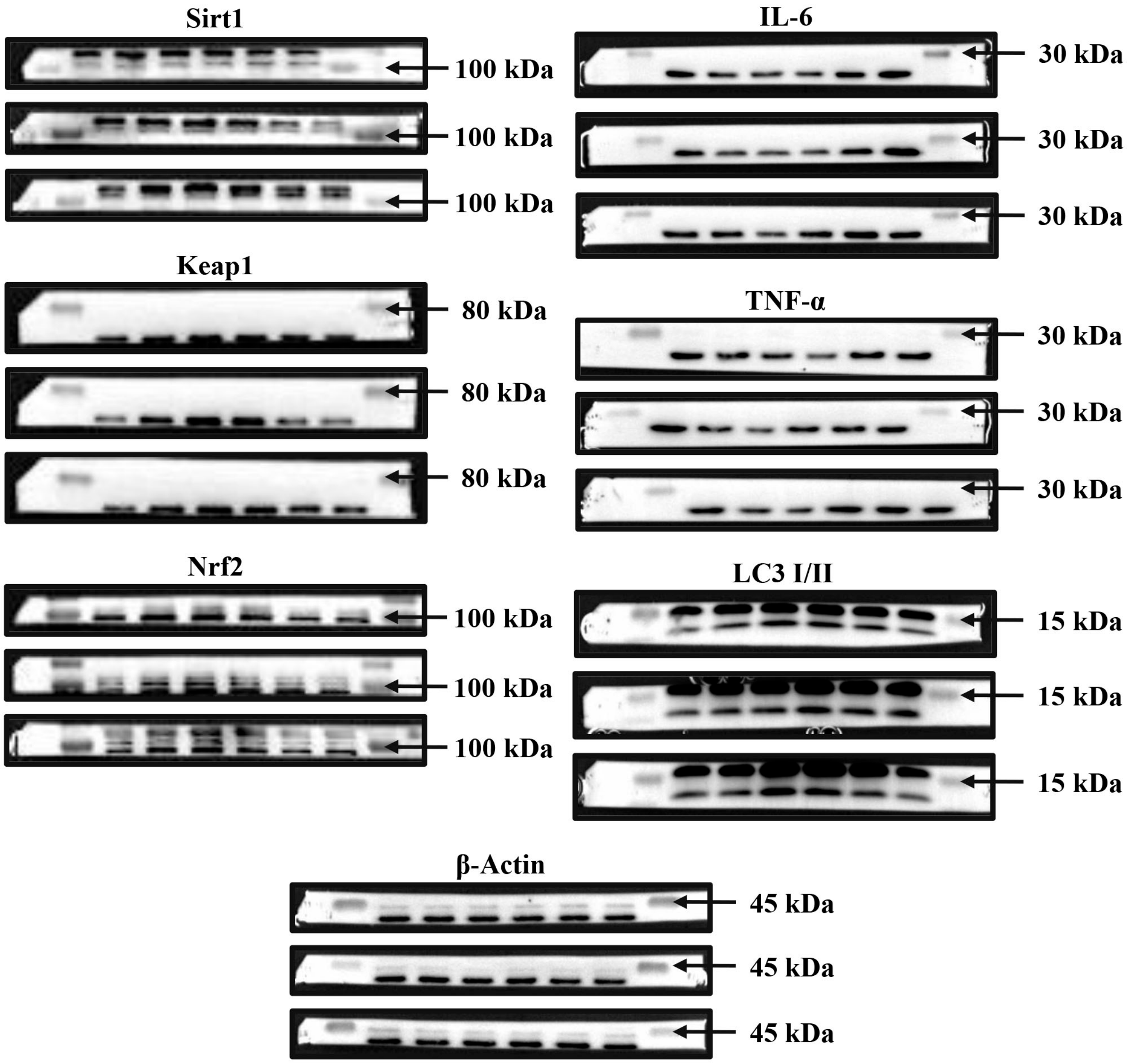

Fig. S1. The uncropped images of Western blots for Fig. 2A-D

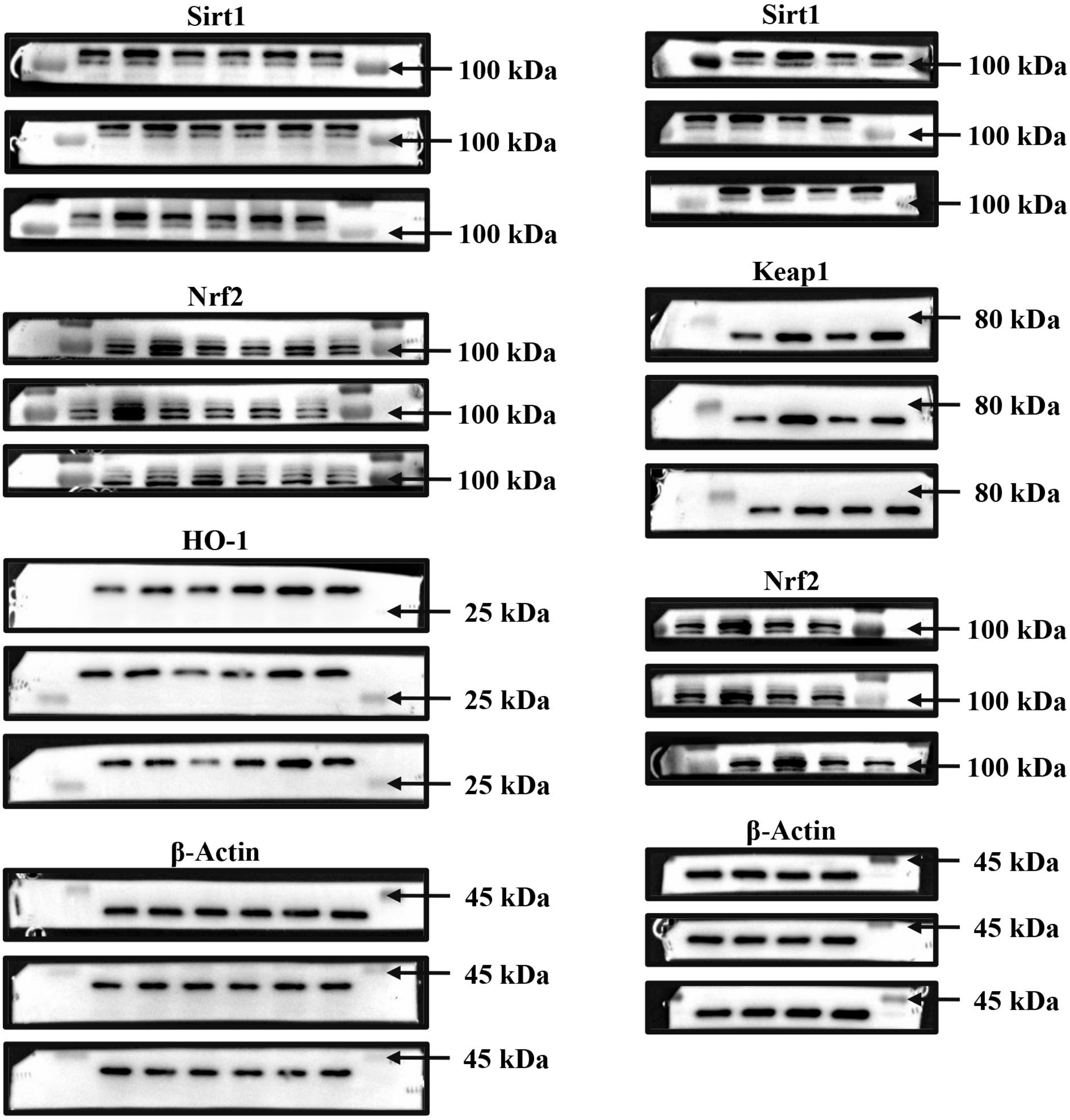

Fig. S2. The uncropped images of Western blots for Fig. 3A-D

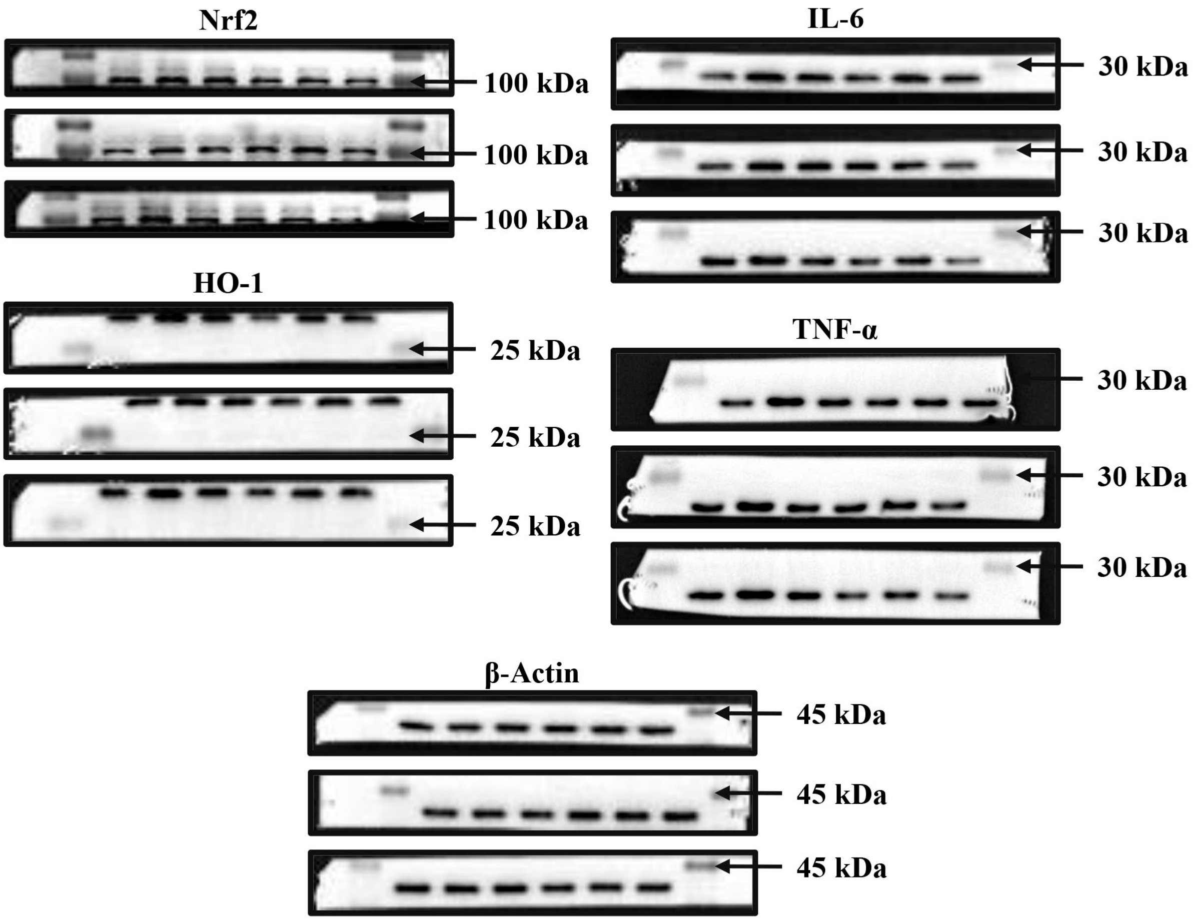

Fig. S3. The uncropped images of Western blots for Fig. 4A-B

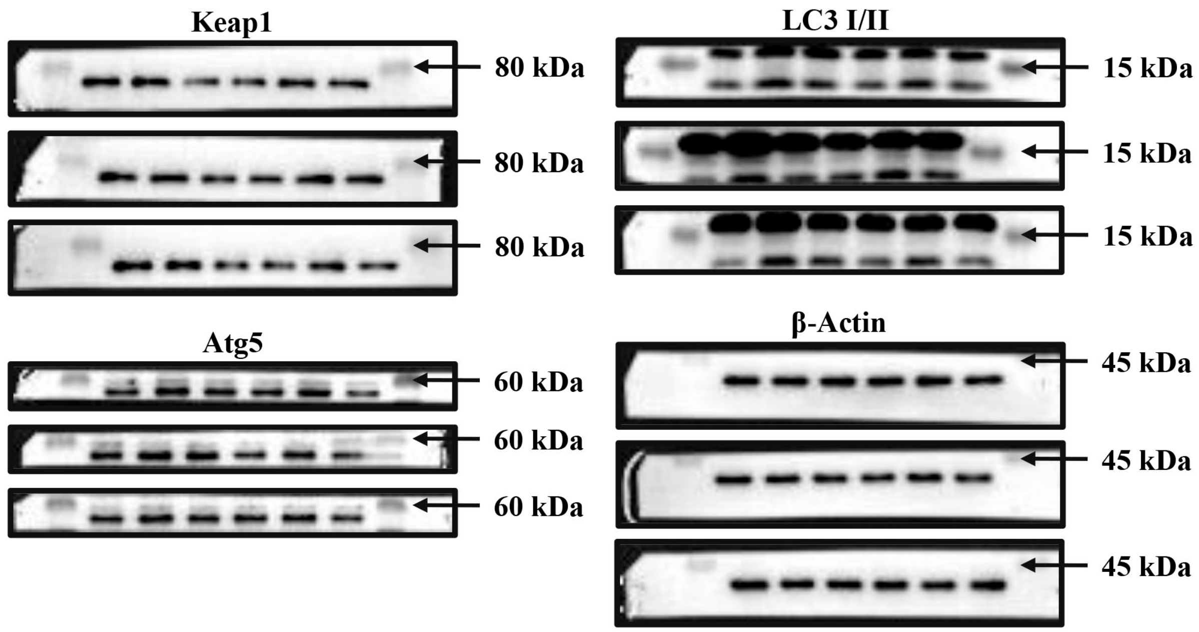

Fig. S4. The uncropped images of Western blots for Fig. 5A-B

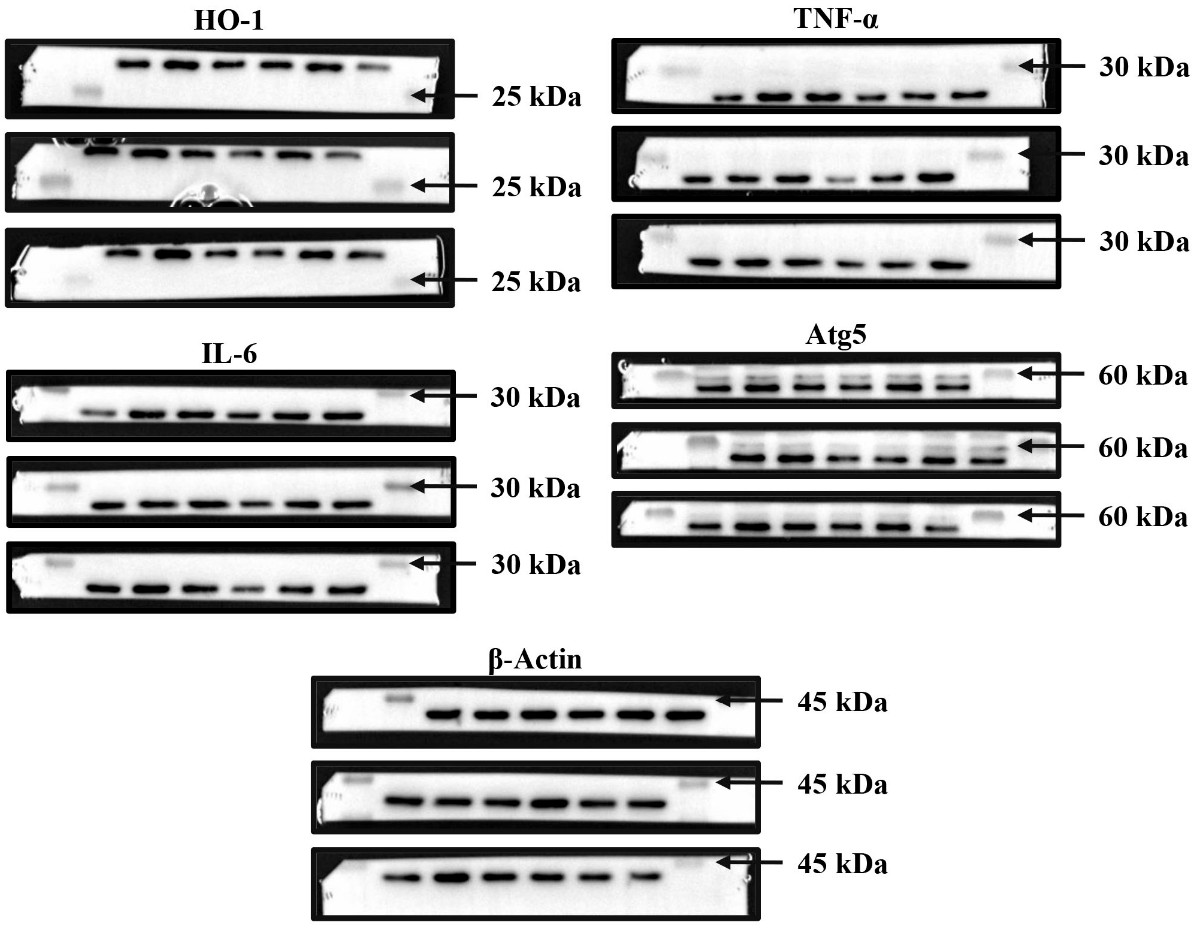

Fig. S5. The uncropped images of Western blots for Fig. 6A-B

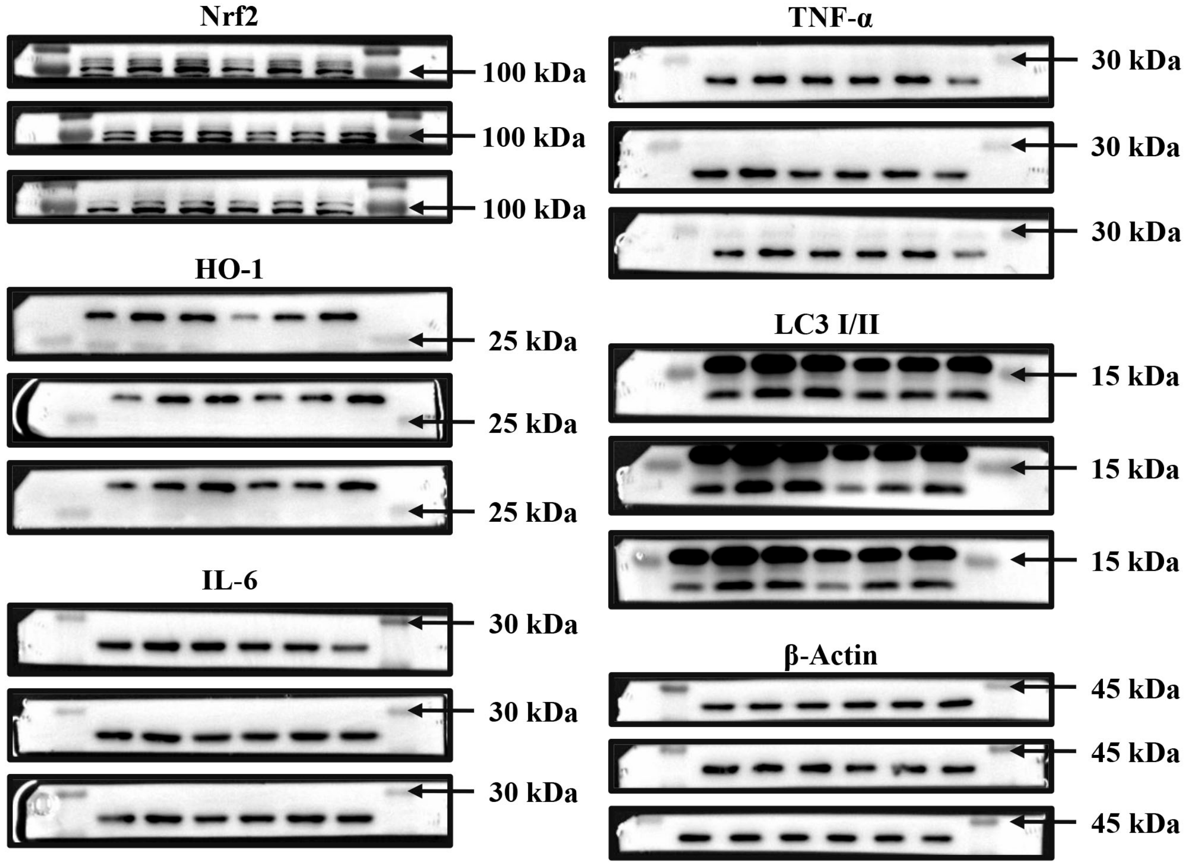

Fig. S6. The uncropped images of Western blots for Fig. 7A-B

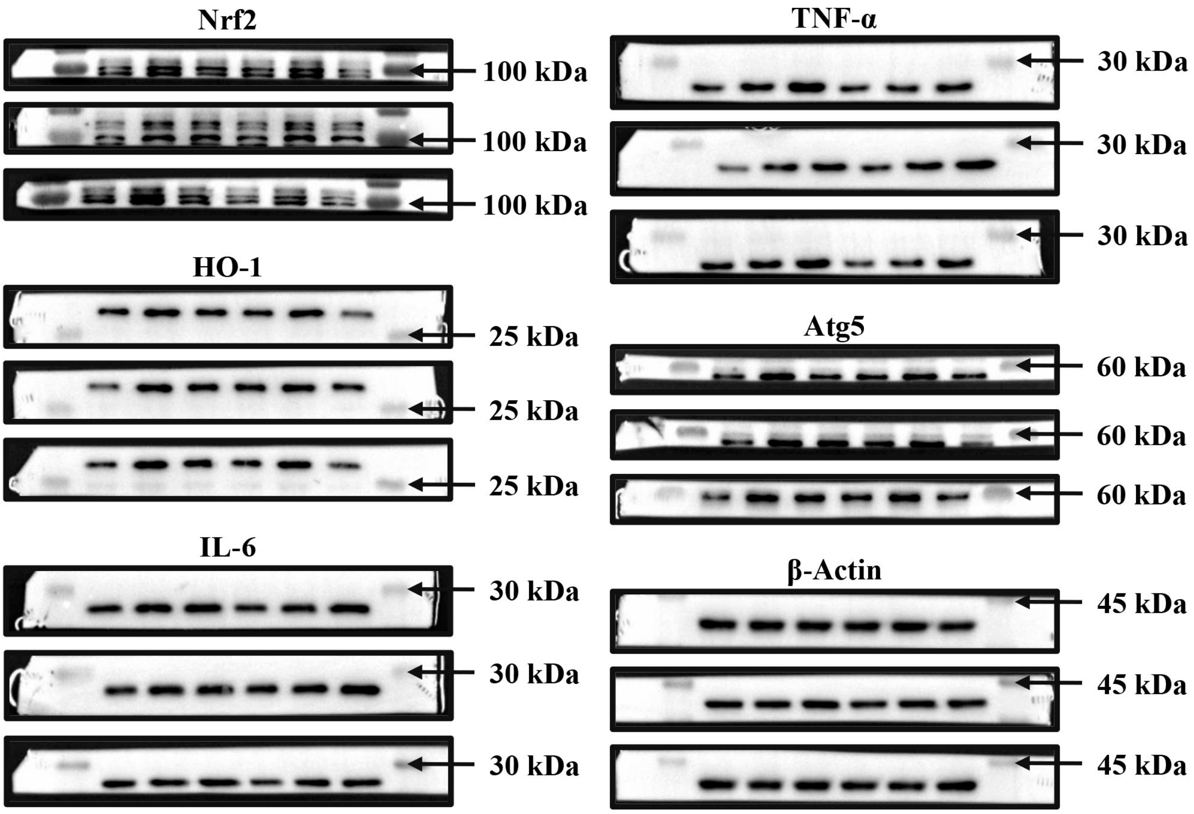

Fig. S7. The uncropped images of Western blots for Fig. 8A-B

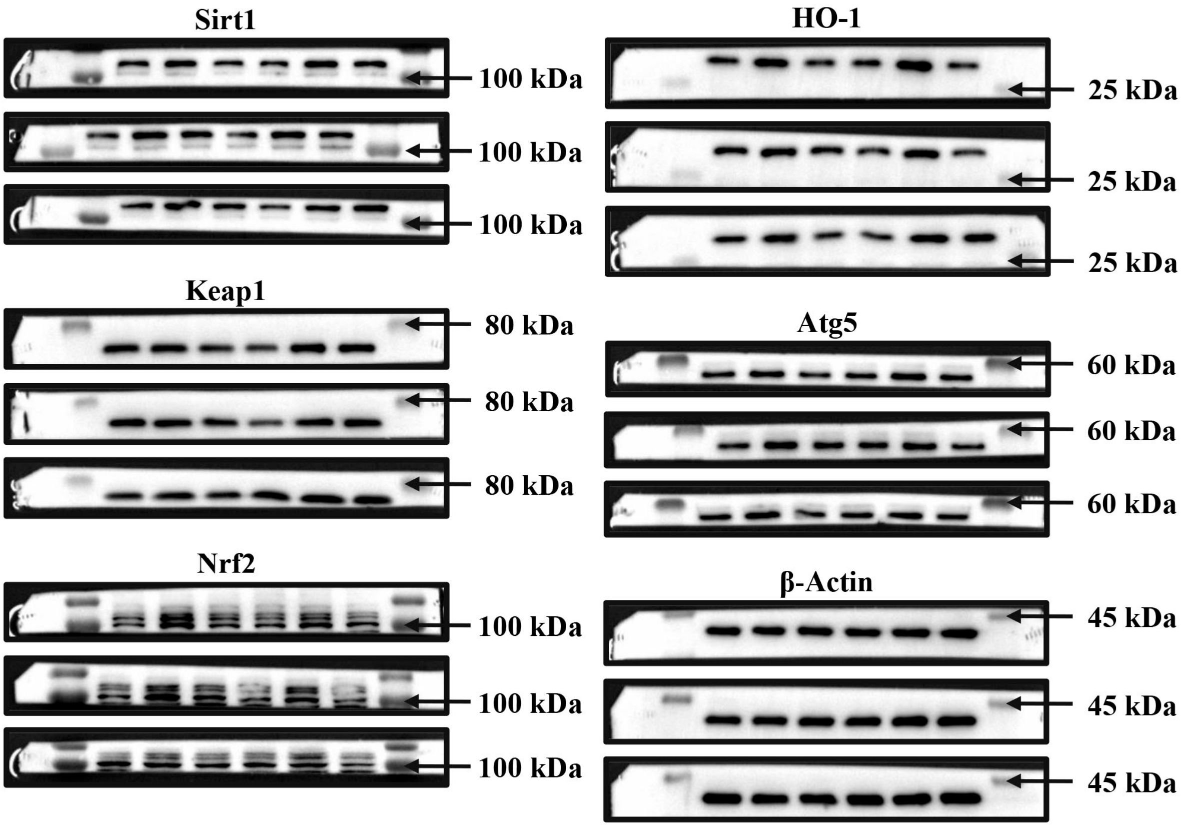

Fig. S8. The uncropped images of Western blots for Fig. 9A-B
